# Genetic and Functional Analyses of Virulence Potential of an *Escherichia coli* O157:H7 Strain Isolated From Super-Shedder Cattle

**DOI:** 10.3389/fcimb.2020.00271

**Published:** 2020-06-05

**Authors:** Lin Teng, Shinyoung Lee, Dongjin Park, Kwangcheol Casey Jeong

**Affiliations:** ^1^Emerging Pathogens Institute, University of Florida, Gainesville, FL, United States; ^2^Department of Animal Sciences, University of Florida, Gainesville, FL, United States; ^3^Food Science and Technology Department, University of Nebraska-Lincoln, Lincoln, NE, United States

**Keywords:** virulence, comparative genomics, super-shedder, functional analysis, *E. coli* O157:H7

## Abstract

Shiga toxin (Stx)-producing *Escherichia coli* (STEC) O157:H7 is an enteric pathogen that causes life-threatening disease in humans, with cattle being major natural reservoirs. A group of STEC O157:H7 with a dramatic combination of high virulence potentials and super-shedder bovine origin have been isolated. Here, an STEC O157:H7 isolate, JEONG-1266, was analyzed by comparative genomics, *stx* genotyping, and phenotypic analyses. The phylogenetic typing and whole-genome comparison consistently showed that JEONG-1266 is genetically close to EC4115 (one of 2006 Spinach outbreak isolates) and SS17 (an isolate from super-shedder cattle) strains, all of which belong to lineage I/II and Clade 8. Both lineage I/II and Clade 8 are known to be mostly associated with clinical strains with high virulence and severe clinical symptoms. Further, JEONG-1266, like EC4115 and SS17, harbors *stx2a/stx2c* genes, and carries Stx-encoding prophages, specifically the φ*stx2a*-γ subtype. Possession of the φ*stx2a*-γ subtype of Stx-encoding prophages and production of Stx2a have been shown to be a key signature associated with hypervirulent STEC O157:H7 strains. *In silico* virulence typing elucidated JEONG-1266, EC4115, and SS17 shared a highly conserved profile of key virulence genes at the nucleotide sequence level. Consistently, phenotypic data showed that JEONG-1266 expressed a high level of Stx2 toxins and had the full capacity of adhesion *in vitro*. Taken together, our study suggests that JEONG-1266 may represent an emerging STEC O157:H7 group, which are hypervirulent strains that originate from super-shedders, that can be a threat to food safety and public health.

## Introduction

Shiga toxin (Stx)-producing *Escherichia coli* (STEC) O157 has become a major public health burden worldwide (Arthur et al., 2013; Munns et al., 2015), especially in Japan, Scotland and North America (Mead et al., 1999; Sakuma et al., 2006; Pollock et al., 2007), since it was first identified as a foodborne pathogen in 1982. *E. coli* O157 infection in humans mainly occurs through consuming food products contaminated with this pathogen (Griffin and Tauxe, 1991; Caprioli et al., 2005; CDC, 2006) and can develop a range of symptoms such as bloody diarrhea and life-threatening hemolytic-uremic syndrome (HUS) (Nataro and Kaper, 1998; Paton and Paton, 1998; Gyles, 2007). During the decade from 2003 to 2012, a total number of 390 *E. coli* O157 outbreaks, including 353 O157 outbreaks, were identified and reported in the United States (Heiman et al., 2015). *E. coli* O157 infections resulted in an average of 63,153 acute cases annually, leading to ~254 million US dollars for the annual cost of illness treatments (Hoffmann et al., 2012).

Cattle are the principal asymptomatic reservoirs of this pathogen (Wells et al., 1991). However, the excretion rate of *E. coli* O157 drastically varies among individual cattle, ranging from 10^2^ to more than 10^7^ CFU/g of feces (Chase-Topping et al., 2007; Jeon et al., 2013). Among these cattle, a subset of them shedding *E. coli* O157 at levels >10^4^ CFU/g in feces are defined as “super-shedders” (Matthews et al., 2006). It has been reported that 20% of most infectious cattle were responsible for about 80% of *E. coli* O157 transmission between animals in Scotch farms (Matthews et al., 2006). Jeon et al. reported super-shedders disseminated *E. coli* O157 to other cattle housed in the same pen, causing the *E. coli* O157 shed by super-shedders predominant in the farm (Jeon et al., 2013). Ecological dynamics of these predominant *E. coli* O157 and super-shedders increase the frequencies of contamination in animal food products such as beef and milk (Gyles, 2007). Hence, it is of great importance to understand the characteristics of the *E. coli* O157 strains from the super-shedders to reduce the prevalence of this pathogen in food-producing animals and consequently to curtail the number of foodborne outbreaks and human illnesses.

The pathogenicity of STEC O157 is determined by its key virulence factors, including Stxs, adhesins, and the type III secretion system (T3SS) and its effectors. Stx, a member of the AB-type toxin family, is the major virulence factor of STEC O157 and uses globotriaosylceramide (Gb3), which is rich in human endothelial cells, as their receptor to gain intracellular translocation (Melton-Celsa, 2014). Stx-medicated cytotoxicity is mainly based on inhibition of protein synthesis on the host target cells by depurinating a specific adenine residue of the 28S rRNA (Croxen et al., 2013). Stxs consist of two major types, Stx1 and Stx2, each of which has variant subtypes. Stx1, Stx2a, and Stx2c, alone or in combination, are the most clinically relevant subtypes. In particular, Stx2a has been shown to be associated with more severe clinical symptoms than Stx1 or Stx2c (Boerlin et al., 1999). The *stx* genes are invariably encoded by a lambdoid prophage in STEC O157 and their production is tightly coupled with phage induction (Tyler et al., 2004). In particular, the level of Stx2 production is a critical virulence parameter for STEC O157 but is known to vary remarkably between STEC O157 strains. A recent study showed that variation in the Stx2 production level in STEC O157 strains is correlated with particular subtypes of Stx-encoding prophages (Ogura et al., 2015).

Intimin and Tir (translocated intimin receptor), encoded by *eae* and *tir* genes, respectively, are a critical adhesin/receptor pair in STEC O157, as they enable the pathogen to intimately bind the intestinal epithelium of the host, resulting in the characteristic attachment and effacement lesion (Melton-Celsa et al., 2012). Several studies with animal models have shown that the intimin/Tir function is essential for stable colonization of STEC O157 in host cells (Mckee et al., 1995; Cornick et al., 2002; Sheng et al., 2006). STEC O157 uses the T3SS, a needle-like structure, to directly translocate Tir and other T3SS effectors inside of the intestinal epithelium of the host. These T3SS effectors, subsequently, manipulate signaling pathways in host cells and establish infection (Melton-Celsa et al., 2012).

STEC O157 have been classified based on several methods including Clade typing (Manning et al., 2008), Lineages classification (Yang et al., 2004), IS-based classification (Stanton et al., 2014), and phylogenetic analysis (Shridhar et al., 2018). Three primary evolutionary lineages of STEC O157 include lineage I (typically from human clinical and bovine sources), lineage II (predominantly from bovine source), and lineage I/II (related to human infection and outbreaks) (Kulasekara et al., 2009; Laing et al., 2009; Stanton et al., 2014). Lineage I/II and Clade 8, in particular, have been shown to be mostly associated with clinical strains with high virulence and severe clinical symptoms. The Stx genotype is one of the conserved characteristics that are associated with those classification schemes; especially, Clade 8 has been proposed to be a highly pathogenic clade of STEC O157 and typically carries *stx2c/stx2a* (Manning et al., 2008; Iyoda et al., 2014).

Besides *in silico* genotyping and comparative genomics, phenotypic and functional characterizations of various key virulence factors are required to fully investigate the virulence potential of STEC O157. Park et al. reported that inactivation of *stx2* gene by the insertion of an IS mobile element can be found naturally in *E. coli* O157 isolates, turning the Shiga toxigenic *E. coli* O157 strain into a non-Shiga toxigenic strain (Park et al., 2013). The same report also showed that an intact *stx* gene encoded in uninducible Stx-prophages was not fully expressed. Therefore, western blotting is necessary to determine the production of intact Stxs in *E. coli* O157 strains. The adhesion of STEC O157 to the human intestinal epithelium is a crucial step for its pathogenicity (Grys et al., 2005).

Despite that STEC O157 from super-shedder cattle have been isolated and investigated in several studies, the complete whole-genome sequences of these isolates are limited in NCBI database and their virulence potential remains unclear. In our previous study, an *E. coli* O157 strain, JEONG-1266, from super-shedder cattle was sequenced using PacBio sequencing method (Teng et al., 2016). In the current study, we performed a comprehensive phylogenetic analysis, comparative genomics, and functional analyses to investigate the virulence potential of JEONG-1266. Our data support that JEONG-1266 may represent a hypervirulent STEC O157 that is associated with super-shedder cattle.

## Materials and Methods

### Bacterial Strains

The *E. coli* O157:H7 strain JEONG-1266 was isolated from the recto-anal junction (RAJ) of a super-shedder cattle located in Florida (Jeon et al., 2013). Genomes of other three *E. coli* O157:H7 strains, EDL933, EC4115, and SS17, were used as reference genomes for comparative analyses. EDL933 (ATCC43895) [NC_CP008957] was linked to a hamburger-borne outbreak in 1982 in the U.S. The clinical isolate, EC4115 [NC_011353], was isolated from a human at the time of 2006 spinach outbreak in Maine, U.S (Eppinger et al., 2011). Another reference strain, SS17 [NZ_CP008805], was collected from the RAJ of super-shedder cattle (Cote et al., 2015).

### Genomic DNA Extraction

Genomic DNA of JEONG-1266 was isolated using QIAGEN DNA mini Kit. Briefly, 1 mL overnight culture of JEONG-1266 was centrifuged at 5,000 × *g* for 10 min. Following the manufacturer's protocol, the bacterial pellet was resuspended in buffer ATL and treated with 20 μL of proteinase K at 56°C for 30 min. The released DNAs were eluted with 100 μL of ddH_2_O.

### Stx2 Subtyping

Polymerase chain reaction (PCR) was performed to determine the *stx2* subtypes (i.e., *stx2a* and *stx2c* gene) carried by JEONG-1266. Two *E. coli* O157:H7 strains, EDL933 carrying *stx2a* and *stx1* and FRIK2455 carrying *stx2c*, were used as controls. Primers used for detecting *stx2a* (stx2a-F: 5′-CTTTTCGACCCAACAAAGTTATGT-3′ and stx2a-R: 5′-CACAGTCCCCAGTATCGCT-3′) and *stx2c* (stx2c-F: 5′-TACTGTGCCTGTTACTGGGC-3′ and stx2c-R: 5′-ACAGTGCCCAGTATCGCC-3′) were designed according to previous study (Jeon et al., 2013). The PCR reaction condition was set as 94°C for 5 min; 94°C for 30 sec, 54°C for 30 sec, 72°C for 60 s for 30 cycles and a final extension time of 10 min at 72°C. Amplified PCR product was analyzed in 1% agarose gel electrophoresis and visualized by ethidium bromide staining.

### Phage Induction

The method of phage induction was previously described (Park et al., 2013). Briefly, cell lysis caused by phage induction was measured by optical density (OD). Overnight culture of bacterial strains was inoculated into Luria-Bertani (LB) broth. Mitomycin C (MMC) treatment (0.5 μg/mL) was followed when the OD_600_ of the cell culture reached 0.7. EDL933 and DH5α were used as a positive and a negative control, respectively, for phage induction. For phage particle preparation, MMC was added to 25 mL of cell culture when OD_600_ reached 0.7 to make a final concentration of 1 μg/mL. After 18 h incubation, unlysed cells and debris were removed by centrifugation at 3,700 × *g* for 20 min at 4°C, and the resultant supernatant was filtered with 0.22 μm-pore-size membrane (Fisher Scientific, USA). Precipitation of phage particles was performed using 0.25 volume of polyethylene glycol/NaCl solution (20% polyethylene glycol 8,000 and 10% NaCl). The polyethylene glycol/NaCl solution containing phage particles was incubated at 4°C overnight, followed by centrifugation at 12,000 × *g* for 1 h. The pellet was resuspended and stored in 1 mL SM buffer (0.58 g NaCl, 0.2 g MgSO_4_·7H_2_O, 0.01 g Gelatin per 100 mL of 1M Tris-Cl pH7.5). SDS-PAGE was used to check the phage protein profile after an aliquot of the phage suspension was mixed with SDS-loading buffer and boiled for 5 min.

### Western Blot

To detect the expression of Stx2, Western blot was conducted as previously described (Jeon et al., 2014). Briefly, the exponential phase culture (OD_600_= 0.7) of an *E. coli* O157 strain was treated with MMC to the final concentration of 1 μg/mL to induce the lytic cycle of phages in the *E. coli* O157 strain and concomitant *stx* gene expression. Next, the O157 cell culture was incubated for 18 h for complete cell lysis. The cell debris was removed by centrifugation (3,700 × *g* for 20 min at 4°C) and the cell-free supernatant was collected by using 0.22 μm-pore-size membrane filter (Fisher Scientific, USA). The total proteins in the supernatant were precipitated by adding 0.25 volume of 100% trichloroacetic acid (Fisher Scientific, USA) to the cell-free culture. The mixture was incubated on ice for 30 min and centrifuged at 12,000 × *g*, 4°C for 30 min. The protein pellet was finally washed using cold acetone. The total proteins were separated by 12% sodium dodecyl sulfate-polyacrylamide gel and transferred to an Immobilon-P polyvinylidene difluoride membrane with a pore size of 0.45 μm (MilliporeSigma, USA) for Western blot. The membrane was blocked using 5% skim milk (BD, USA) in TBST (10 mM Tris-HCL [pH 7.4], 150 mM NaCl, and 0.05% Tween-20) at room temperature for 1 h. Later, the membrane was incubated overnight at 4°C with monoclonal Verotoxin II-a subunit antibody (Meridian Life Science Inc., USA), and washed using TBST at room temperature for 1 h. The membrane was then incubated with HRP conjugated secondary antibody (GE Healthcare, USA) diluted 1:10,000 in TBST, washed with TBST, and incubated with a chemiluminescent substrate (GE Healthcare, USA). Finally, the membrane was exposed to Kodak BioMax film (Sigma, USA).

### Adherence Assay to HEp-2 Cells

The ability of JEONG-1266 to adhere to human epithelial type-2 (HEp-2) cells was evaluated. EDL933 was used as a positive control and DH5α was used as a negative control. HEp-2 cells were maintained in Dulbecco's Modified Eagle Medium (DMEM; Coring, USA) composed of 10% (vol/vol) heat-inactivated fetal bovine serum at 37°C and 5% CO_2_. Approximately 1 × 10^5^ HEp-2 cells were seeded into a 24-well polystyrene plate as measured using a hemocytometer and allowed to grow until 90% confluence. Bacterial cultures were grown overnight at 37°C in LB broth and washed with sterile phosphate-buffered saline (PBS) three times. The final cell pellet was resuspended in PBS to a final concentration of OD_600_ = 1 (5 × 10^8^ CFU/mL). An aliquot (20 μL) of the resuspended culture was added into 480 μL of DMEM. Then 500 μL of DMEM containing 1 × 10^7^ CFU of bacteria was added to each well to obtain a multiplicity of infection (MOI) of 100. After a 3-h incubation at 37°C with 5% CO_2_, the bacterial suspension was removed and 500 μL of new DMEM was added to each well for another 3-h incubation. The infected cell monolayer was washed using sterile PBS three times to remove any unattached bacteria. To detach the HEp-2 cells from the plate, 1 mL of 0.1% Triton X-100 in PBS buffer was added to each well and allowed to incubate for 5 min. The media was collected from each well, serially diluted in LB broth, and plated on LB agar plates to enumerate the number of attached bacterial cells. The adherence assay of each strain was conducted in triplicate. Statistical significance between the level of attachments of isolates was accessed using a student's *t*-test (α = 0.05).

### Comparative Genomics

The complete genome of JEONG-1266 was acquired using PacBio sequencing and annotated by NCBI annotation pipeline (Teng et al., 2016). The completed genome was circularly presented by CGview (Stothard and Wishart, 2005; Overbeek et al., 2014; Brettin et al., 2015). All the coding sequences (CDSs) were assigned to different subsystems based on their functions using RAST (Overbeek et al., 2014; Brettin et al., 2015). Mauve 2.3.1 with default setting (Darling et al., 2004, 2010) was employed to compare the whole-genome architecture of JEONG-1266 and reference strains. Pangenome analysis was conducted to identify unique genes among strains using Prokka annotation and Roary (Seemann, 2014; Page et al., 2015). To identify the position of prophage related genes, the PHAge Search Tool (PHAST) (http://phast.wishartlab.com/) (Zhou et al., 2011) with default setting was used. The positions of predicted prophage regions of JEONG-1266, as well as reference genomes, were presented using BLAST Ring Image Generator (BRIG) (Alikhan et al., 2011). To investigate the similarity of Stx2a-encoding prophages in JEONG-1266 and other three reference strains, the nucleotide sequences of the prophages were compared using BLASTn embedded in EasyFig (Sullivan et al., 2011). Genes encoding known and putative virulence factors in the genome of JEONG-1266 were identified using the Virulence Factor Database (VFDB) (Chen et al., 2016) in Pathosystems Resource Integration Center (PATRIC) (https://www.patricbrc.org/portal/portal/patric/Home) (Wattam et al., 2014). The false-negative virulence genes were manually curated by doing BLASTn against whole-genome sequences of each isolate. The multilocus sequence typing (MLST) of *E. coli* can be identified using two different sets of housekeeping genes (Wirth et al., 2006; Jaureguy et al., 2008). Both typing methods were employed by MLST 2.0 in the Center for Genomic of Epidemiology (http://www.genomicepidemiology.org/).

### *In silico* Genotyping

*In silico* genotyping was used to predict the pathogenicity of JEONG-1266. Two methods were used to identify the clade of JEONG-1266. An *in silico* Clade 8 *rhsA* was used to determine whether the isolate belongs to the Clade 8 based on the presence of a SNP (3468C) in the *rhsA* gene (Liu et al., 2009). Besides, another *in silico* analysis based on 32 SNPs was also conducted to determine the clade of JEONG-1266 (Manning et al., 2008). To identify LSPA-6 profile of JEONG-1266, sequences of six pairs of primers were applied for *in silico* PCR (Yang et al., 2004). An isolate showing an LSPA-6 profile of 111111 was defined as lineage I. A strain with LSPA-6 profile of 222222 or 222223 was defined as lineage II. Other genotypes were defined as lineage I/II (Stanton et al., 2014). The subtype of Stx2-encoding prophage was identified by BLAST the sequence of PCR primers against the nucleotide sequence of the Stx2-encoding prophage (Ogura et al., 2015).

### Phylogenetic Analysis

A phylogenetic tree of 26 *E. coli* O157: H7 strains was constructed using Parsnp (Treangen et al., 2014). The numbers of SNPs between strains were calculated using the VCF file generated by Parsnp. Figtree (v.1.4.2) (http://tree.bio.ed.ac.uk/software/figtree/) was used to edit the phylogenetic tree.

### Accession Numbers

The complete genome sequence of *E. coli* O157 strain JEONG-1266 [NZ_CP014314], EDL933 [NC_CP008957], EC4115 [NC_011353], and SS17 [NZ_CP008805] were deposited in NCBI. The accession numbers of the strains used for comparative genomics were listed in the supplementary data (Table S1).

## Results

### A Genomic Overview of JEONG-1266

**Figure 1:**
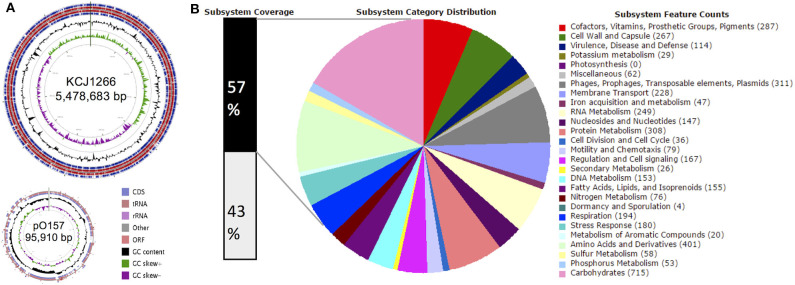
Genomic overview. **(A)** Genome map of JEONG-1266. The chromosome (top) and plasmid (bottom) of JEONG-1266 were displayed. Marked characteristics are shown from outside (ring 1) to the center (ring 6): Coding Sequences (CDSs), tRNA and rRNA in forward strand (ring1), Open Reading Frames (ORFs) in forward strand (ring 2); ORFs in reward strand (ring 3); CDSs, tRNA and rRNA in reward strand (ring 4), GC content (ring 5), and GC skew (ring 6). **(B)** Subsystem category distribution. The annotated genes in JEONG-1266 were classified into different subsystem categories based on gene function. The subsystems contain 57% of total genes (black bar on the left). The other 43% of genes (white bar on the left) do not belong to any of the subsystems.

The STEC O157:H7 strain JEONG-1266 contained a chromosome of 5,478,683 and a 95,910 bp pO157 plasmid, with a GC content of 50.5% (Figure 1A, Table 1). Its chromosome was shown to encode for 5,363 CDSs, 106 tRNAs, and 22 rRNAs, while the pO157 contained 100 genes. Compared to the three reference strains of STEC O157:H7 including a super-shedder strain (SS17) and two outbreak-associated strains (EDL933 and EC4115), the chromosome of JEONG-1266 was slightly shorter in size, containing fewer CDSs (Table 1). The number of rRNA genes was highly conserved, while that of tRNA genes differed between the four strains, with the highest tRNA gene number in JEONG-1266. Functional categorization of genes by RAST assigned 57% (3,082 of 5,363 CDSs) of genes with the 27 subsystems categories, leaving the other 43% of the genes unclassified (Figure 1B). Among the classified genes, 114 genes belonged to the category of virulence, disease, and defense (Figure 1B), which was focused in this study.

**Table 1:** Genome statistics of JEONG-1266 and reference O157 strains.

	**JEONG-1266**	**SS17**	**EC4115**	**EDL933**
Length of sequence (Mb)	5.48	5.52	5.57	5.53
G+C ratio (%)	50.5	50.5	50.5	50.4
Coding sequence (CDS)	5,363	5,389	5,488	5,435
No. rRNA	22	22	22	22
No. tRNA	106	103	105	101
Plasmid (bp; gene number)	pO157 (95910; 100),	pO157 (94,645;100), pSS17 (37,447; 43)	pO157 (94644;101), pEC4115 (37452; 43)	pO157 (92076;95)

### Phylogenetic Characterization of the JEONG-1266 Strain

**Figure 2:**
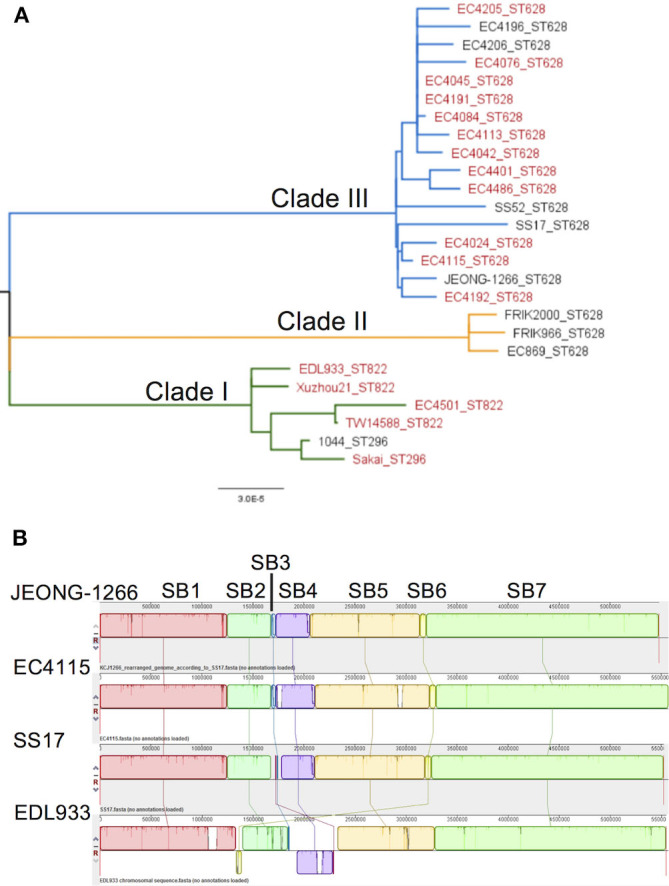
Phylogeny and genomic architecture of JEONG-1266 and other *E. coli* O157 strains. **(A)** Phylogenetic relatedness of JEONG-1266 with other *E. coli* O157 strains. A maximum-likelihood phylogenetic tree was constructed based on the core-genome SNPs of 26 *E. coli* O157 strains from a variety of sources (Table S1). All the strains clustered into three major clades, i.e., Clade I (green branches), Clade II (orange branches), and Clade III (blue branches). The strains associated with outbreaks are in red. **(B)** Mauve alignments of JEONG-1266 with three reference genomes. Alignments of JEONG-1266 with the three reference strains (SS17, EC4115, and EDL933) reveal 7 synteny blocks (SB) with the size ranging from ~45.4 kb to ~ 2,276 kb. Each block is represented by a different color with a line connecting to its homologous SB on the chromosome of other strains. The white regions indicate the non-homology region.

SNP-based phylogenetic analysis of JEONG-1266 was performed using the core genome sequences of 26 previously characterized *E. coli* O157:H7 strains isolated from diverse sources (Table S1). The 26 isolates clustered into three major clades, Clade I, II, and III (Figure 2A). Clade I contained clinical strains that have been associated with outbreaks and severe diseases in humans, while Clade II consisted of strains of bovine origin (FRIK966 and FRIK2000) and ground beef (EC869) (Figure 2A and Table S1). Clade III was composed of both bovine and clinical strains (Figure 2A and Table S1). JEONG-1266 belonged to Clade III, which consisted of 2006 multistate spinach outbreak strains (e.g., EC4115) (Eppinger et al., 2011) and the super-shedder bovine strains (e.g., SS17) (Cote et al., 2015). In particular, the SNP matrix data revealed that JEONG-1266 is most closely related to EC4115 (48 SNPs) and EC4191 (56 SNPs) among the spinach outbreak strains (Figure S1). The MLST profile showed that all of the Clade II and Clade III strains analyzed, including JEONG-1266, were typed as ST628 (Figure 2A). Four strains in Clade I (EDL933, Xuzhou21, TW14588, and EC4501) belonged ST822, while the other 2 Clade I strains (Sakai and 1044) displayed a different MLST type, *i.e*., ST296 (Figure 2A). Therefore, SNP-based phylogeny and MLST together support that JEONG-1266 strain is closely related with STEC O157:H7 from two particular sources; super-shedder bovine and the spinach outbreak.

### The Genome Architecture and Prophages

To further understand the genomic characteristics of JEONG-1266, the whole-genome architecture of JEONG-1266 and the other 3 reference strains (SS17, EC4115, and EDL933) were analyzed using Mauve 2.3.1. JEONG-1266 and EC4115 have 7 synteny blocks (SBs), while SS17 and EDL933 have 8 SBs. The EDL933 genome contained inverted SB4 and SB6 (Figure 2B). In addition, an extra non-homology block was found in SS17 and EDL933. Consistent with the phylogenetic analysis results, the genome architecture of JEONG-1266 was most similar to that of EC4115.

*E. coli* O157:H7 genomes are characterized by the presence of multiple prophages whose sequences are highly variable among strains. Many non-phage genes with virulent functions are present in prophages, including *stx* and *tccP* (an effector for LEE T3SS) (Garmendia et al., 2005). PHAST analysis identified a total of 19 prophages, including 11 intact prophages, in JEONG-1266 (Table 2, Figure S2). When the chromosome sequences of JEONG-1266 was compared with that of EC4115 and SS17, it carried 20 and 79 unique genes, respectively (Table S2). These unique genes located in prophage regions, mostly encoding hypothetical proteins or phage-related proteins. Furthermore, genomic variations between JEONG-1266 and the reference strains were mainly detected in prophage regions, P1, P4, P8, P9, and P10 (Table 2, Figure 2B). Except for P10, all the other four variable prophages were predicted to be intact. The genome of JEONG-1266 was shown to have two intact Stx-encoding prophages, P12 and P14, each of which carried *stx* subtypes, *stx2c* and *stx2a*, respectively.

**Table 2:** Genome encoded in the 19 phage regions of JEONG-1266.

**Phage regions**	**Size (Kbp)**	**Completeness**	**Synteny block**	**Encoded genes**
P1	26.5	Intact	SB1	Phage DNA invertase; Phage integrase; and phage assembly protein
P2	38.5	Intact	SB1	Lysozyme and Attachment invasion locus protein precursor
P3	38	Incomplete	SB1	Phage assembly proteins and 3 tRNAs
P4	29.4	Intact	SB1	Attachment invasion locus protein precursor and phage lysis
P5	50.5	Intact	SB2	Integrase and phage assembly proteins
P6	46.5	Intact	SB2	Integrase, phage lysin, attachment invasion locus protein precursor
P7	8	Questionable	SB2	Mobile element proteins
P8	57	Intact	SB2 and SB3	Attachment invasion locus protein precursor, 2 tRNAs, phage lysis, *acfC*, and integrase
P9	94.8	Intact	SB4 and SB5	Attachment invasion locus protein precursor, phage lysis 5 tRNAs, and integrase
P10	24.8	Incomplete	SB5	Integrase and 3 tRNAs
P11	54	Intact	SB5	Integrase, phage lysin, *inv* and 5 tRNAs
P12	61.2	Intact	SB5	Attachment invasion locus protein precursor, integrase, *yeeV, yeeX, stx2*, and 3 tRNAs
P13	24	Intact	SB5	Phage assembly proteins and Phage lysin
P14	71.7	Intact	SB5	Integrase, attachment invasion locus protein precursor, and *stx2*
P15	77.2	Questionable	SB5, SB6, and SB7	Integrase, attachment invasion locus protein precursor, Phage lysin, and 4 tRNAs
P16	23.9	Questionable	SB7	Phage lysin and integrase
P17	13.1	Questionable	SB7	Mobile element proteins
P18	20.5	Questionable	SB7	Integrase, *yeeU, yeeV, espA, espB, espD, espF, sseE, yscL, escD, eae*, and *tir* chaperone
P19	21.7	Questionable	SB7	Integrase and phage *eae*

### Analysis of Virulence Genes

**Figure 3:**
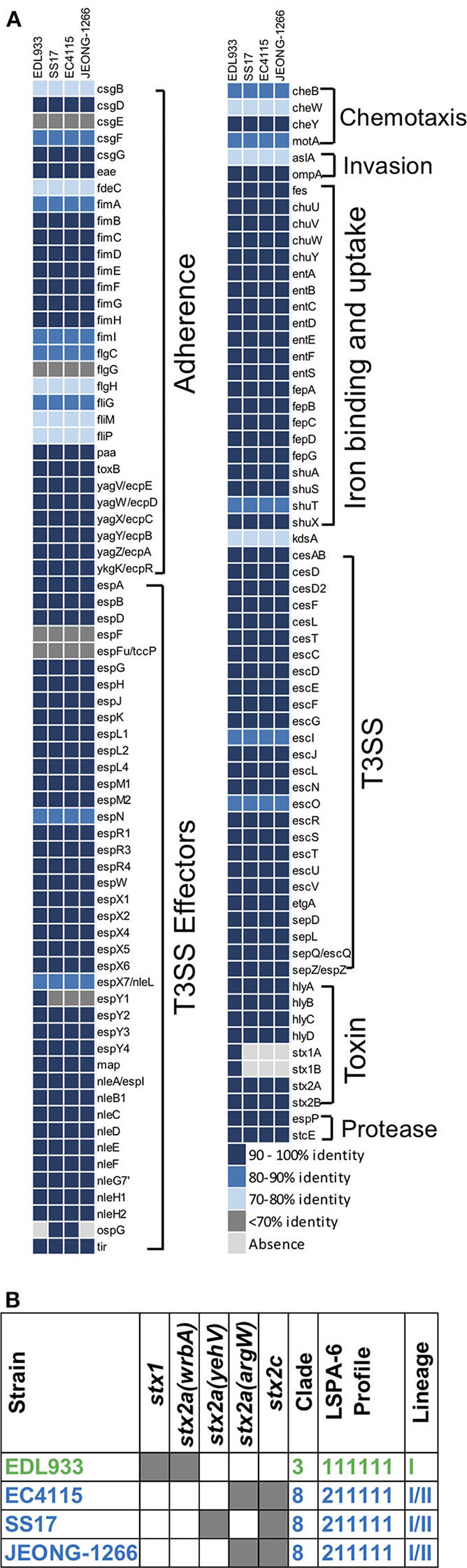
Virulence gene profiles and genotyping of *E. coli* O157. **(A)** Virulence gene profile of JEONG-1266 and other *E. coli* O157 strains, SS17, EC4115, and EDL933. The virulence genes of 4 strains were identified by aligning their CDSs against the protein sequences in VFDB by BLASTp. Compared with the sequences of virulence genes in VFDB, virulence genes in 4 strains showed distinct identities that were displayed using 5 color codes. **(B)** Genotyping of *stx* subtypes, clade, and lineage of JEONG-1266 and other STEC O157:H7 strains. A shaded gray box indicates the presence of the *stx* genes, and the insertion loci of the Stx2-encoding prophage.

Besides *stx* genes, the presence of other virulence genes was analyzed to address the virulence potential of the JEONG-1266 strain. In the four strains, a total of 132 virulence genes were identified using VFDB in PATRIC and categorized into 8 major functional groups: adherence (30 genes), chemotaxis (4 genes), invasion (2 genes), iron binding and uptake (21 genes), toxin (8 genes), protease (2 genes), T3SS (26 genes), and T3SS effectors (40 genes) (Figure 3A). Eight toxin genes were characterized; *hlyABCD* for hemolysin, *stx1AB* for Stx1, and *stx2AB* for Stx2. JEONG-1266, SS17, and EC4115 strains shared a highly conserved virulence profile overall. Most of the virulence genes displayed high homology (>90%) to the reference genes in the VFDB (Figure 3A). The most divergent gene categories (<90%) to the database include adherence and chemotaxis. The adherence-associated genes included intimin (*eae*), cytotoxin (*toxB*), outer membrane adhesin Paa (*paa*), *E. coli* common pilus (*ecpABCDER*), curli (*csgBEFG*), type 1 fimbriae (*fimABCDEFGHI*), and flagellar (*flgCGH and fliGMP*). JEONG-1266 encoded conserved T3SS structural genes as well as a total of 41 putative T3SS effector genes, indicating the presence of a functional T3SS. The essential genes of the locus of enterocyte effacement (LEE) (Nataro and Kaper, 1998), including *tir, eae, espA*, and *espB*, were shown to be highly conserved. Taken together, these results indicate that JEONG-1266 possesses an array of virulence genes required for the full virulence potential of STEC O157, a characteristic that often lacks in strains of bovine origin.

### Determination of Lineage, Clade, and *stx* Genotype

Combinational characteristics of the lineage type (I, II, I/II), *stx* genotype, the insertion site of the Stx-encoding prophage have been shown to be conserved in STEC O157 and useful to determine virulence potential and evolutionary relationship of the pathogen (Stanton et al., 2014). To determine the lineage of JEONG-1266, *in silico* LSPA-6 typing was performed using nucleotide sequences of the 6 polymorphism markers (Yang et al., 2004). JEONG-1266 showed a lineage I/II profile (i.e., 211111) (Figure 3B), which is identical to those of SS17 and EC4115 (Stanton et al., 2014; Cote et al., 2015).

Clade determination was conducted based on 32 SNP loci on the chromosome of *E. coli* O157:H7 (Manning et al., 2008). The resultant SNP profile indicated that EDL933 belonged to the Clade 3 while JEONG-1266, EC4115, and SS17 belonged to the Clade 8 (Figure 3B; Table S3). The Clade 8 has been shown to be the most virulent clade of *E. coli* O157:H7 due to high levels of Stx expression and cytotoxicity (Neupane et al., 2011).

Two types of Stx, Stx1, and Stx2, show distinct cytotoxicity and Stx2 is more frequently associated with severe diseases in humans than Stx1 (Melton-Celsa, 2014). In addition, variable Stx2 subtypes have been identified, including Stx2a and Stx2c, and Stx2a is known to be more toxic to humans than Stx2c (Schmitt et al., 1991). Stx2a and Stx2c are typically encoded in different Stx-encoding prophages. *In silico* analysis showed that both *stx2a* and *stx2c* are present in JEONG-1266, EC4115, and SS17 but only JEONG-1266 and EC4115 shared the same insertion site for Stx2a-encoding prophage, the *argW* locus (Figure 3B). SS17 and EDL933 used *yehW* and *wrbA* loci as the insertion site, respectively. These results suggest that the Stx2a-encoding prophage of JEONG-1266 is more related to that of EC4115.

Taken together, combinational genetic characteristics of JEONG-1266 (*i.e.*, the lineage/clade types, *stx* genotype, and the insertion site of the Stx-encoding phage) further support its high virulence potential, especially being a member of the hypervirulent Clade 8 with the *stx2a*/*stx2c* genotype.

### Characterization of Stx2a-encoding Prophage Subtype

Of the two types of Stx2, Stx2a and Stx2c, Stx2a is known to as a major risk factor for severe STEC infections. Ogura et al. identified four major subtypes of Stx2a-encoding prophage (α, β, γ, and δ) and found that strains carrying φs*tx2a-*γ or φ*stx2a*-α (particularly the former) can produce higher Stx2 levels than strains carrying other *stx2a* phage subtypes (Ogura et al., 2015).

**Figure 4:**
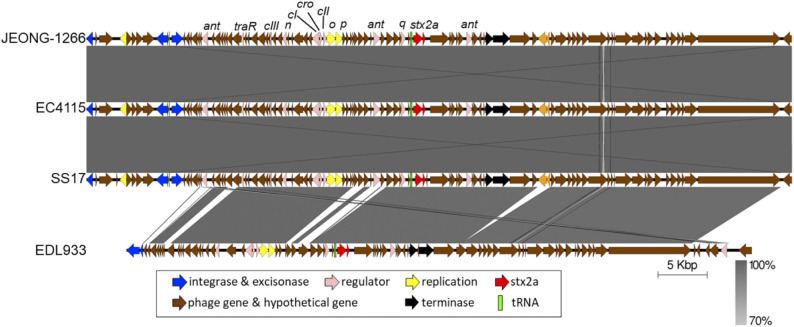
Comparison of Stx2a-encoding prophages. The Stx2a-encoding prophage of JEONG-1266 was compared with the ones in EC4115, SS17, and EDL933. The CDS and tRNA were shown in colored arrows and blocks, respectively. The darkness of gray shade between strains reflects the similarity of the nucleotide sequences of Stx2a-encoding prophages.

The subtype of Stx2a-encoding prophage of JEONG-1266 was determined by *in silico* PCR analysis, using the nested primer sets designed for Stx2a-encoding prophage subtyping (Ogura et al., 2015). The results showed that Stx2a-encoding prophage carried by JEONG-1266 is φ*stx2a-*γ. Since both EC4115 and EDL933 have been shown to carry φ*stx2a-*γ (Ogura et al., 2015), the genome structures of the Stx2a-encoding prophage were compared among JEONG-1266, SS17, EC4115, and EDL933. The length of the Stx2a-encoding prophage in JEONG-1266 (71,736 bp), SS17 (71,730 bp), and, EC4115 (71,736 bp) were highly similar, while it was shorter in EDL933 (63,575 bp) (Figure 4). Next, the nucleotide sequences of the entire prophage genomes were compared by BLASTn. The similarity of the Stx2a-encoding prophages was as follows; 99.98% for JEONG-1266 vs. SS17 (100% coverage, 17 nucleotides difference, and 6 gaps), 100% for JEONG-1266 vs. EC4115 (100% coverage, 1 nucleotide difference, and 0 gaps), and 97.60% for JEONG-1266 vs. EDL933 (74% coverage) (Table S4). Stx2a-encoding prophage in JEONG-1266, EC4115, and SS17 have 102 CDSs, while the one in EDL933 has 83 CDSs. Therefore, *in silico* PCR typing and prophage genome analysis together showed that JEONG-1266, SS17, and EC4115 are highly likely lysogenized by the same φ*stx2a-*γ, indicating their strong potential to produce high levels of Stx2 toxins. Our data showed that the Stx2a-encoding prophage of EDL933 is structurally different from that of JEONG-1266, SS17, and EC4115, although it was determined to be φ*stx2a-*γ subtype.

### Shiga Toxin Production

**Figure 5:**
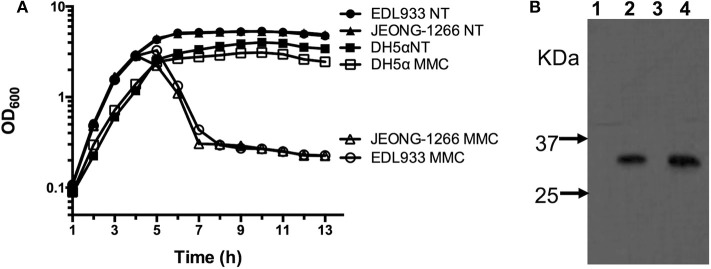
Analyses of Shiga toxin production. **(A)** Survival curve of JEONG-1266, as well as EDL933 and DH5α, with (MMC) and without (NT) mitomycin C treatment. Mitomycin C was added to bacterial cell cultures when the OD_600_ reached 0.7. **(B)** Western blotting to detect the expression of Shiga toxin 2 in JEONG-1266 with and without mitomycin C treatment. EDL933 was used as a positive control. Samples from Lane 1 to 4 were EDL933 without MMC (lane 1), EDL933 with MMC (lane 2), JEONG-1266 without MMC (lane 3), and JEONG-1266 with MMC (lane 4).

Stx production of JEONG-1266 was analyzed by MMC-mediated induction of the Stx-encoding prophages. The EDL933 strain was used as a positive control and DH5α, a *stx* gene-negative strain, was used as a negative control. The OD_600_ value of both JEONG-1266 and EDL933 decreased after MMC treatment, while the growth of DH5α was not affected by MMC treatment (Figure 5A). This result indicates that bacterial cells of JEONG-1266 and EDL933 were lysed by phage induction.

To investigate the production of Stx2 toxins by JEONG-1266, we conducted Western blot using Stx2-specific monoclonal antibodies in the presence and absence of MMC. Both JEONG-1266 and EDL933 produced Stx2 toxins with MMC dependency (Figure 5B); whether the toxins were Stx2a and/or Stx2c was not determined in this experiment since the Stx2-specific monoclonal antibody does not have differential specificities for Stx2a and Stx2c.

### Adhesion of JEONG-1266

**Figure 6:**
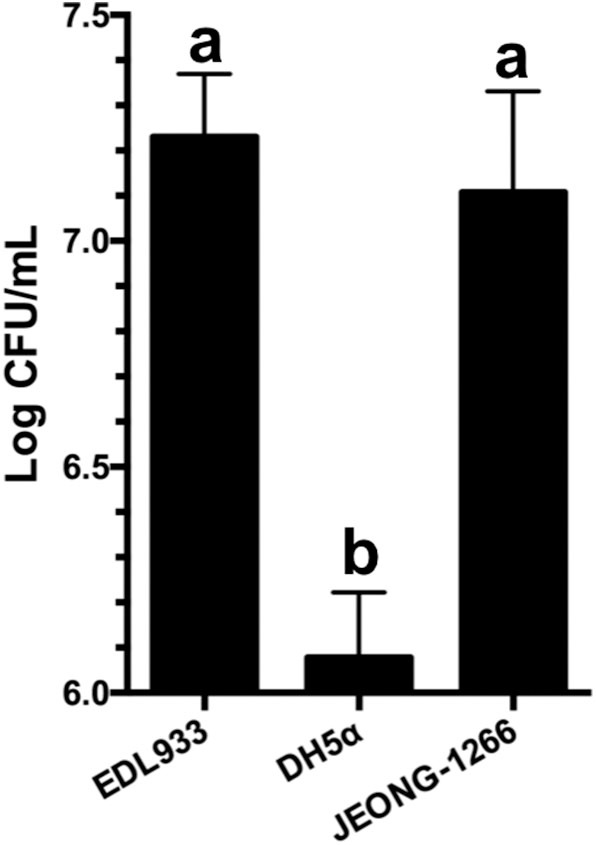
Adherence of JEONG-1266 to HEp-2 cells. The adherence capability of JEONG-1266 to HEp-2 cells was compared with EDL933 (positive control) and DH5α (negative control). The assay was performed in triplicate. Statistical analysis was conducted using a student's *t*-test (α = 0.05). Means with different letters differ significantly.

Adherence of the STEC O157 to the epithelial cells of the human intestinal tract is the initial step for its colonization and pathogenesis. STEC O157 EDL933 strain has been extensively studied in its ability to adhere to and colonize the human intestinal tract (Lewis et al., 2015; Cordonnier et al., 2017). To evaluate the adherence capability of JEONG-1266 to human cells, adherence assay was conducted using human HEp-2 cells with EDL933 and DH5α as a positive and negative control, respectively. JEONG-1266 showed a significant increase in adherence to the HEp-2 cells (*P* < 0.05) compared with DH5α (Figure 6). The adherence capability of JEONG-1266 was similar with that of EDL933 (*P* > 0.05), suggesting that JEONG-1266 is comparable to EDL933 in its ability to colonize the human intestinal tract.

## Discussion

To investigate the potential impact of an *E. coli* O157:H7 isolate which was a super-shedder origin on humans, we performed comparative genomics, phylogenetic analysis, and functional analyses and characterize the virulence potential. Phylogenetic analysis with 26 *E. coli* O157 strains from various sources showed that JEONG-1266 belongs to the prominent cluster including all spinach outbreak strains and super-shedder strains (Clade III, Figure 2A). In particular, JEONG-1266 formed a subclade with EC4115, a clinical isolate from the 2006 spinach outbreak, and SS17, one of two super-shedder strains analyzed. The subsequent comparative analysis by Mauve revealed the significant collinearity in the whole-genome architecture between JEONG-1266, EC4115, and SS17 (Figure 2B). The genome of JEONG-1266 was shown to contain a total of 19 prophage regions. Despite prophage regions of *E. coli* O157:H7 genomes are typically hypervariable, those 19 prophage regions were shown to be highly conserved among the three strains, implying their close evolutionary relationships. This result strongly indicates the presence of an ecological connection between *E. coli* O157:H7 strains from super-shedders and clinical strains from the outbreak, which can represent a potential control point to prevent preharvest-stage transmission of *E. coli* O157.

Manning et al. identified the presence of hypervirulent subgroup, called Clade 8, of *E. coli* O157 associated with severe clinical symptoms such as HUS (Manning et al., 2008). Our data showed that JEONG-1266, together with EC4115 and SS17, belongs to Clade 8, highlighting the importance of the Clade 8 of bovine-origin. Clade 8 strains typically produce high levels of Stx toxins and are lysogenized with particular Stx-encoding prophage subtypes (Ogura et al., 2015). To further understand the Stx-related genetic characteristics of JEONG-1266, *stx* genotype and Stx2a-encoding prophage are analyzed. The finding that JEONG-1266, EC4115, and SS17 are lysogenized with the same subtype of Stx2a-encoding prophage, φ*stx2a-*γ, implies that these strains have similar Stx-medicated cytotoxicity in humans despite their diverse origin (*i.e*., super-shedder cattle and clinical isolation). As the expression of the *stx2* genes is directly controlled by the lambdoid late promoter of the Stx2-encoding prophage (Waldor and Friedman, 2005), it is plausible that the subtype of an Stx2-encoding prophage is a critical determinant for Stx2 production levels. It is worth note, however, that Stx2-encoding prophage subtypes (α, β, γ, and δ) are classified by their well-conserved genome structure, their gene content can be variable within each subtype (Ogura et al., 2015), indicating in-depth genetic resolution of Stx2-encoding prophage may help better understanding of Stx production and virulence potential of *E. coli* O157:H7.

The whole genomes analysis revealed that genome-wide architecture (Figure 2B) and virulence gene profile (Figure 3A) are highly conserved among JEONG-1266, EC4115, and SS17 as well. This result is consistent with the previous study showing the similarity between SS17 and EC4115 genomes (Cote et al., 2015). There were only 48 SNPs in the core genome of JEONG-1266 in comparison to EC4115 (Figure S1). Both our phylogenetic data and the previous studies (Cote et al., 2015; Katani et al., 2017) clearly showed that SS17 and SS52 from super-shedders are phylogenetically closed to EC4115. As expected, JEONG-1266, SS17, and EC4115 strains shared a highly conserved virulence profile overall (Figure 3A); the most divergent gene categories (<90% to the VFDB) include adherence and chemotaxis, both of which are typically related to the host specificity and environmental interactions. Consistently, our phenotypic analysis showed that JEONG-1266 displayed high capability in adherence to HEp-2 cells. Similar results were also observed by Cote et al. (2015) and Katani et al. (2015) when they evaluated the adherence capability of STEC O157:H7 from super-shedders using bovine RAJ stratified squamous epithelial (RSE) cells. Those STEC O157:H7 from super-shedder cattle, including SS17 and SS52, formed aggregates surrounding the RSE cells. Given their genomic and genetic similarities, JEONG-1266 may also have a similar super-shedder phenotype. Most importantly, the virulence profile result indicates that JEONG-1266 possesses a comprehensive array of virulence genes required for the full virulence potential of STEC O157:H7, a characteristic that often lacks in strains of bovine origin.

Besides its cytotoxicity, Stx2 has been implicated in enhancing adherence to and colonization of intestinal epithelial cells by *E. coli* O157:H7, possibly by increasing the expression of the host cell receptor for the pathogen (Robinson et al., 2006; Liu et al., 2010). Furthermore, the presence of *stx2a* was epidemiologically associated with the super-shedder of the pathogen by cattle (Matthews et al., 2013). Most recently, Fitzgerald el al. demonstrated that Stx2a has a critical role in animal-to-animal transmission of STEC and the development of super-shedders (Fitzgerald et al., 2019). Our data showed that JEONG-1226, together with EC4115 and SS17, carries *stx2a* and the Stx2a-encoding prophage subtype, φ*stx2a-*γ, suggesting that the occurrence of such STEC subgroup in human clinical isolates and outbreaks may arise as a result of high cattle shedding levels associated with the *stx* genetic properties. Together, a dramatic combination of the super-shedder phenotype and hypervirulence potential of JEONG-1266 revealed by this study highlights the epidemiological and clinical importance of the STEC O157:H7 group of bovine origin.

## Conclusion

In summary, the genetic and functional analyses revealed that *E. coli* O157:H7 strain JEONG-1266, isolated from super-shedder cattle belongs to hypervirulent Clade 8, produces Stx2, shows high adherence capability, and contains major virulence genes typically present in clinical isolates. The hypervirulence potential of JEONG-1266 was also supported by the genome-wide phylogenetic and SNP analysis as well as high-resolution subtyping of Stx-encoding prophage of the strain. An emerging subgroup of STEC O157:H7 with a combination of hypervirulence and super-shedder imposes a new threat for the public health and food industry. A better understanding of bacterial genetics and host ecology of the specific subgroup of STEC may help us control the transmission of the pathogen at the pre-harvest level.

## Data Availability Statement

The datasets generated for this study can be found in the NCBI/NZ_CP014314/, https://www.ncbi.nlm.nih.gov/nuccore/NZ_CP014314.1.

## Ethics Statement

The animal study was reviewed and approved by University of Florida Institutional Animal Care and Use Committee (IACUC number 201003744).

## Author Contributions

LT, SL, DP, and KJ designed the study and drafted the manuscript. LT and SL performed the analyses. LT, DP, and KJ finalized the manuscript. KJ acquired funding.

## Conflict of Interest

The authors declare that the research was conducted in the absence of any commercial or financial relationships that could be construed as a potential conflict of interest.
