# Supplementary material for: Genetic and Functional Analyses of Virulence Potential of an Escherichia coli O157:H7 Strain Isolated From Super-Shedder Cattle
Source: Front Cell Infect Microbiol. 2020 Jun 5;10:271. doi: 10.3389/fcimb.2020.00271 (PMC7289925; doi:10.3389/fcimb.2020.00271)
Supplement: Supplementary file 1 [file Table_1.DOCX]

Table S1. Background information of 26 strains used in this study.

| **Strain name** | **Outbreak** | **Source** | **Origin** | **LSPA** | **Accession #** |
| --- | --- | --- | --- | --- | --- |
| EDL933 | Ground hamburger outbreak | Human | MI | Lineage I | CP008957 |
| Xuzhou21 | Xuzhou outbreak | Human | Xuzhou, China | Lineage I | CP001925 |
| TW14588 | Taco John outbreak | Lettuce | MI | Lineage I | [ABKY00000000](http://www.ncbi.nlm.nih.gov/nuccore/ABKY00000000) |
| EC4501 | Taco John outbreak | Human | MN | Lineage I | [ABHT00000000](http://www.ncbi.nlm.nih.gov/nuccore/ABHT00000000) |
| Sakai | Sakai outbreak | Human | Sakai, Japan | Lineage I | BA000007 |
| 1044 | Reference strain | NA* | NA | Lineage I | [AERP00000000](http://www.ncbi.nlm.nih.gov/nuccore/AERP00000000) |
| EC869 | Reference strain | Ground beef | PA | Lineage II | [ABHU00000000](http://www.ncbi.nlm.nih.gov/nuccore/ABHU00000000) |
| FRIK966 | Bovine reservoir | Feces | WI | Lineage II | [ACXN00000000](http://www.ncbi.nlm.nih.gov/nuccore/ACXN00000000) |
| FRIK2000 | Bovine reservoir | Feces | FL | Lineage II | [ACXO00000000](http://www.ncbi.nlm.nih.gov/nuccore/ACXO00000000) |
| EC4205 | Spinach outbreak | Bovine | CA | Lineage I/II | [ADVB00000000](http://www.ncbi.nlm.nih.gov/nuccore/ADVB00000000) |
| EC4191 | Spinach outbreak | Spinach bag | IL | Lineage I/II | [ADVA00000000](http://www.ncbi.nlm.nih.gov/nuccore/ADVA00000000) |
| EC4084 | Spinach outbreak | Human | WI | Lineage I/II | [ADUY00000000](http://www.ncbi.nlm.nih.gov/nuccore/ADUY00000000) |
| EC4192 | Spinach outbreak | Human | CA | Lineage I/II | [ADUX00000000](http://www.ncbi.nlm.nih.gov/nuccore/ADUX00000000) |
| EC4206 | Bovine reservoir | Bovine | CA | Lineage I/II | [ABHK00000000](http://www.ncbi.nlm.nih.gov/nuccore/ABHK00000000) |
| EC4045 | Spinach outbreak | Spinach bag | NM | Lineage I/II | [ABHL00000000](http://www.ncbi.nlm.nih.gov/nuccore/ABHL00000000) |
| EC4042 | Spinach outbreak | Human | NM | Lineage I/II | [ABHM00000000](http://www.ncbi.nlm.nih.gov/nuccore/ABHM00000000) |
| EC4113 | Spinach outbreak | Spinach bag | UT | Lineage I/II | [ABHP00000000](http://www.ncbi.nlm.nih.gov/nuccore/ABHP00000000) |
| EC4196 | Bovine reservoir | Bovine | CA | Lineage I/II | [AKMC00000000](http://www.ncbi.nlm.nih.gov/nuccore/AKMC00000000) |
| EC4076 | Spinach outbreak | Human | WI | Lineage I/II | [ABHQ00000000](http://www.ncbi.nlm.nih.gov/nuccore/ABHQ00000000) |
| EC4024 | Spinach outbreak | Human | OH | Lineage I/II | [ABJT00000000](http://www.ncbi.nlm.nih.gov/nuccore/ABJT00000000) |
| EC4486 | Taco Bell outbreak | Human | NJ | Lineage I/II | [ABHS00000000](http://www.ncbi.nlm.nih.gov/nuccore/ABHS00000000) |
| EC4401 | Taco Bell outbreak | Human | PA | Lineage I/II | ABHR00000000 |
| SS17 | Bovine reservoir | Super-shedder Bovine | Midwestern United States | Lineage I/II | [CP008805](http://www.ncbi.nlm.nih.gov/nuccore/CP008805.1,CP008806.1,CP008807.1) |
| SS52 | Bovine reservoir | Super-shedder Bovine | Midwestern United States | Lineage I/II | [CP010304](http://www.ncbi.nlm.nih.gov/nuccore/CP010304.1,CP010305.1) |
| EC4115 | Spinach outbreak | Human | ME | Lineage I/II | [CP001164](http://www.ncbi.nlm.nih.gov/nuccore/CP001164,CP001165,CP001163) |
| JEONG-1266 | Bovine reservoir | Super-shedder Bovine | FL | Lineage I/II | [CP014314](http://www.ncbi.nlm.nih.gov/nuccore/CP014314) |

*NA: information not available.
