# Supplementary figures and images for: Genetic and Functional Analyses of Virulence Potential of an Escherichia coli O157:H7 Strain Isolated From Super-Shedder Cattle
Source: Front Cell Infect Microbiol. 2020 Jun 5;10:271. doi: 10.3389/fcimb.2020.00271 (PMC7289925; doi:10.3389/fcimb.2020.00271)

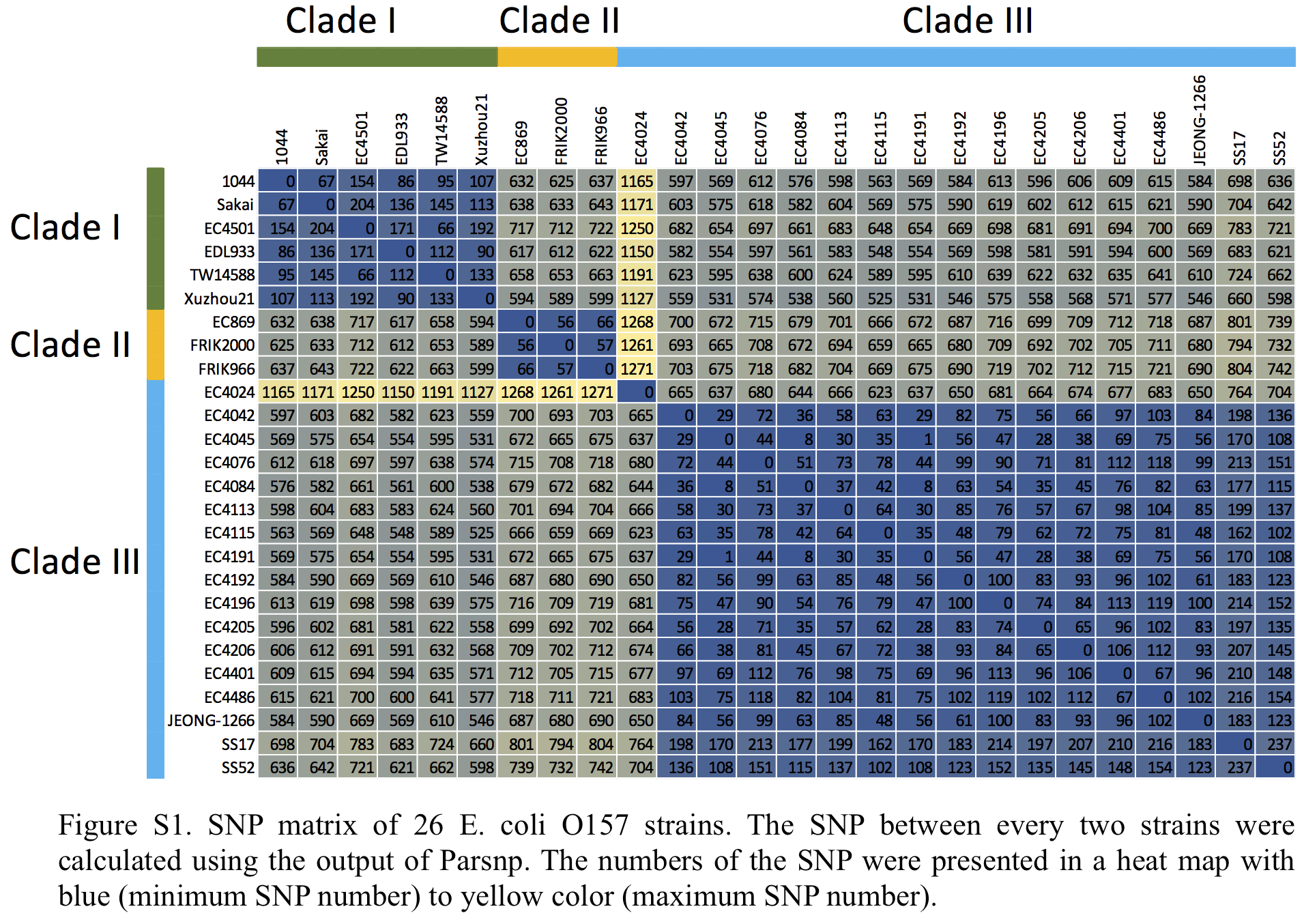

Supplement: Supplementary file 5 [file Image_1.TIFF]

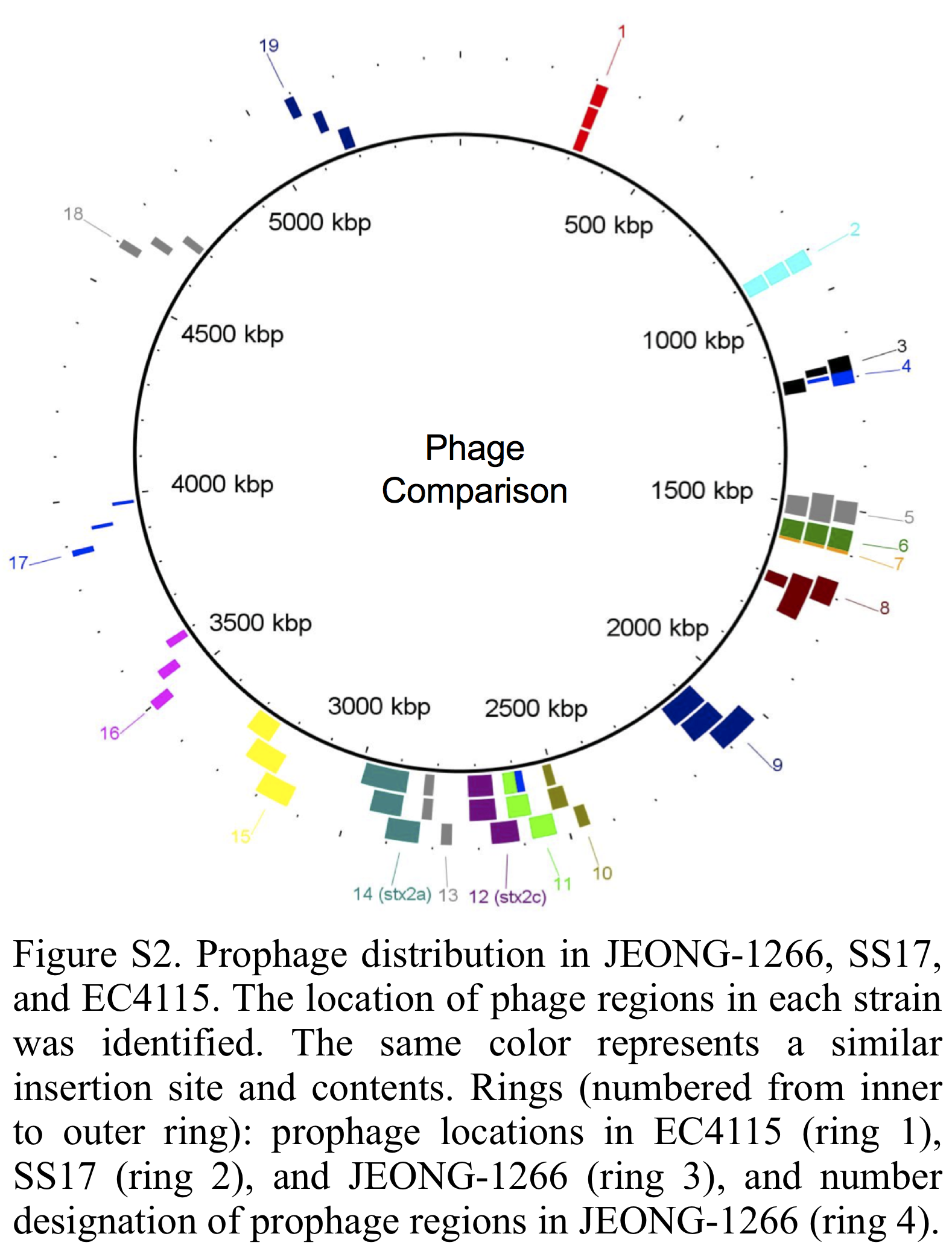

Supplement: Supplementary file 6 [file Image_2.TIFF]
